# Advances in the prevention and treatment of Alzheimer’s disease based on oral bacteria

**DOI:** 10.3389/fpsyt.2023.1291455

**Published:** 2023-12-13

**Authors:** Miaomiao Zhang, Nannan Mi, Zheng Ying, Xiaoping Lin, Ying Jin

**Affiliations:** Department of Stomatology, Shengjing Hospital of China Medical University, Shenyang, China

**Keywords:** Alzheimer’s disease, oral-gut-brain axis, early diagnosis, dental prophylaxis, anti-bacterial agents, probiotics, vaccine, healthy lifestyle

## Abstract

With the global population undergoing demographic shift towards aging, the prevalence of Alzheimer’s disease (AD), a prominent neurodegenerative disorder that primarily afflicts individuals aged 65 and above, has increased across various geographical regions. This phenomenon is accompanied by a concomitant decline in immune functionality and oral hygiene capacity among the elderly, precipitating compromised oral functionality and an augmented burden of dental plaque. Accordingly, oral afflictions, including dental caries and periodontal disease, manifest with frequency among the geriatric population worldwide. Recent scientific investigations have unveiled the potential role of oral bacteria in instigating both local and systemic chronic inflammation, thereby delineating a putative nexus between oral health and the genesis and progression of AD. They further proposed the oral microbiome as a potentially modifiable risk factor in AD development, although the precise pathological mechanisms and degree of association have yet to be fully elucidated. This review summarizes current research on the relationship between oral bacteria and AD, describing the epidemiological and pathological mechanisms that may potentially link them. The purpose is to enrich early diagnostic approaches by incorporating emerging biomarkers, offering novel insights for clinicians in the early detection of AD. Additionally, it explores the potential of vaccination strategies and guidance for clinical pharmacotherapy. It proposes the development of maintenance measures specifically targeting oral health in older adults and advocates for guiding elderly patients in adopting healthy lifestyle habits, ultimately aiming to indirectly mitigate the progression of AD while promoting oral health in the elderly.

## 1 Introduction

The human oral cavity possesses a multifaceted and intricate microbial ecosystem, creating a distinctive microenvironment housing the oral microbiome, which stands as the second most prevalent microbial community following the gastrointestinal tract (1). The oral microbiome is composed of an assemblage of bacteria, fungi, and viruses, encompassing a rich diversity of approximately 1,000 bacterial species (2) that can be classified into six primary phyla: *Actinobacteria*, *Bacteroidetes*, *Firmicutes*, *Proteobacteria*, *Spirochaetes*, and *Fusobacteria* (3). Although the vast majority of these microorganisms are characterized as non-pathogenic, a minor subset possesses the capability to assume the role of opportunistic pathogens. Through intricate interactions among themselves and the host, these microorganisms function in tandem to maintain the delicate ecological balance, thwarting colonization by exogenous pathogens, and fostering not only oral but also systemic well-being. Nevertheless, disturbances in the dynamic equilibrium of the oral microbiota can engender various oral disorders, notably periodontitis (4) and dental caries, which ultimately undermine the normal physiological functions of the oral cavity. Furthermore, oral bacteria harbor the potential to translocate to distant organs, instigating chronic inflammatory responses and may potentially play a contributory role in the genesis of systemic ailments, such as Alzheimer’s disease.

Alzheimer’s disease (AD) is a chronic neurodegenerative disorder characterized by progressive memory impairment and cognitive impairment, making it the most prevalent form of dementia among the elderly (5). With the global population continuing to age, there has been a noticeable increase in the prevalence of AD (6). It is estimated that approximately 55 million individuals worldwide are affected by dementia, and projections indicate that this number will rise to 78 million by 2030 (7). Regarding the financial burden, global AD treatment costs are anticipated to account for 17.52, 18.71, 20.00, 20.80, and 20.70% of total expenditures in 2015, 2020, 2030, 2040, and 2050, respectively (8). These statistics underscore the substantial economic and societal burdens associated with AD. Despite extensive research efforts and some insight into its pathogenesis, the precise etiology of AD remains elusive, and there is currently a lack of effective strategies for prevention and treatment. Consequently, AD continues to have a high mortality rate, ranking as the seventh leading cause of death globally in 2019 (9).

## 2 The relationship between oral microbiota and AD

### 2.1 Epidemiological relevance

In 1891, Miller, a prominent figure in the realm of oral microbiology, introduced the concept of “oral focal infection” (10). This paradigm proposed that the oral microbiota possesses the capacity to trigger infections in remote anatomical sites, thereby contributing to the development of various systemic diseases. Miller’s seminal hypothesis served as a cornerstone in establishing the intrinsic connection between oral health and general well-being, subsequently guiding further investigations in the field of oral microbiology.

AD and periodontitis exhibit shared risk factors and demonstrate notable similarities in their heightened inflammatory profiles, thus suggesting a potential bidirectional relationship (11). Furthermore, the oral microbiota, serving as the instigating factor for periodontitis, emerges as a crucial link between periodontal disease and AD, particularly with periodontal pathogens assuming significant roles in the mechanisms underlying AD onset (12). The density of oral bacteria in the brains of individuals with AD is estimated to be approximately sevenfold higher compared to cognitively healthy individuals (13). Furthermore, research has demonstrated that alterations occur in the oral microbiota of AD patients, and periodontal microbiota is relatively sensitive to cognitive changes (14). Importantly, this heightened density encompasses elevated levels of periodontal pathogens such as *Porphyromonas gingivalis* and *Fusobacterium nucleatum* (15, 16), in addition to *Prevotella intermedia* (15) and *Treponema denticola*, commonly found in dental plaque (17). Substantial evidence points to an association between alterations in the bacterial composition of the oral microbiota, elevated levels of inflammatory cytokines, and AD (15). In tandem, the dental and periodontal health of AD patients gradually declines as the disease progresses, intricately interwoven with their cognitive function (18). When the depth of periodontal pockets exceeds 6 mm, the risk of developing AD escalates by a remarkable 15-fold (19).

Undesirable oral conditions, encompassing tooth loss and inadequate oral hygiene practices, can exert profound effects on the composition and diversity of oral bacteria, thereby potentially predisposing individuals to cognitive decline (20). Notably, AD patients exhibit an increased risk of tooth loss and complete edentulism, surpassing the risks observed within the general population (21). A prospective cohort study has revealed a longitudinal association between the number of teeth present (NTP) and hippocampal atrophy, particularly when considering the severity of periodontitis, and this underscores the potential link between tooth loss and subsequent cognitive decline, which may outweigh the influence of age (22). Moreover, the loss of functional teeth and impairment of functional occlusal units can further exacerbate cognitive impairments in affected individuals (23).

A prospective longitudinal study involving 5,468 participants revealed a robust link between infrequent toothbrushing routines and the occurrence of AD (24). Furthermore, with advancing age, individuals diagnosed with AD often encounter difficulties in adequately maintaining their oral hygiene practices (25), resulting in a pronounced decline in oral health status (26). Consequently, dental professionals are increasingly recognizing the importance of identifying and addressing risk factors associated with oral health while promoting regular oral care practices.

**Figure 1:**
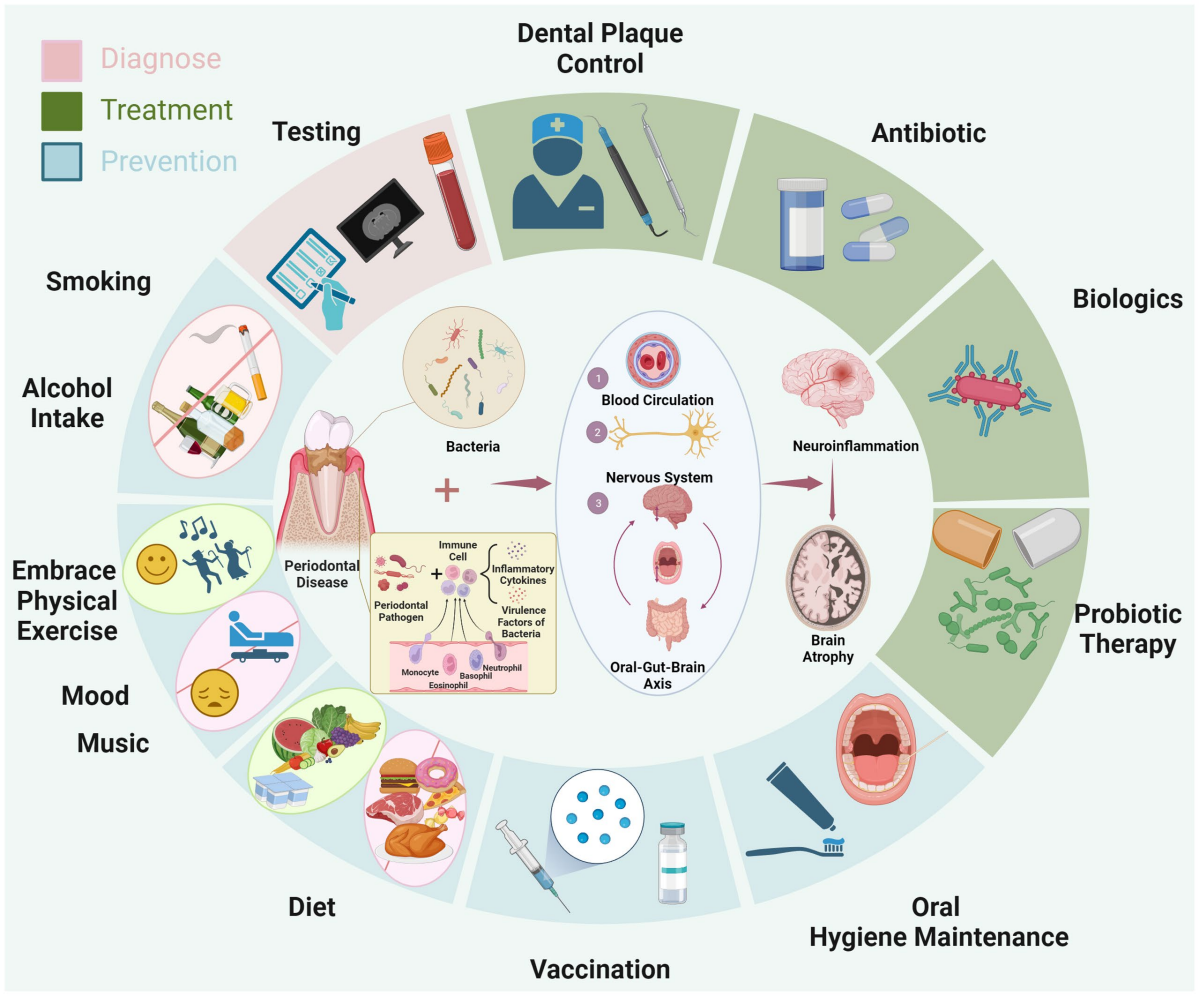
The diagram illustrates the mechanism through which periodontitis contributes to AD. Specifically, periodontal pathogens lead to chronic inflammation in oral tissues, triggering the influx of inflammatory and bacterial virulence factors into the bloodstream. These substances circulate through the blood, nervous system, and the oral-gut-brain axis, eventually causing chronic inflammation in the brain. This ultimately promotes the progression of AD. Surrounding the diagram, in a circular manner, are depicted the approaches for early diagnosis (pink area), treatment (green area), and prevention of AD (blue area) based on the aforementioned pathological mechanism. Created with BioRender.com.

### 2.2 Pathological mechanism relevance

Certainly, AD is a multifactorial and intricate condition, driven by various factors, with primary hypotheses including the amyloid cascade hypothesis (27) and the neuroinflammatory hypothesis (28). The neuropathological characteristics associated with these hypotheses encompass the abnormal accumulation of extracellular amyloid-β (Aβ) and the entanglement of intraneuronal neurofibrillary tangles with hyperphosphorylated tau protein. These features collectively constitute the complex disease mechanism of AD. However, the outcomes of recent drug trials targeting the amyloid cascade hypothesis have generated less encouraging findings, casting doubts on the effectiveness of treatment strategies associated with this hypothesis (29). Consequently, the neuroinflammation hypothesis and Tau hyperphosphorylation has garnered attention as a focal point of current investigations within the realm of AD research.

The dysregulated composition of the oral microbiota exhibits a close association with detrimental oral health outcomes and neuroinflammation, thereby initiating neurodegeneration (30, 31). Specifically, oral microorganisms produce various virulence factors, including gingipains (32), lipopolysaccharides (33), and outer membrane vesicles(OMVs) (34), which subsequently mediate chronic periodontal inflammation and damage to the periodontal support tissues. It is noteworthy that research has shown that OMVs produced by periodontal pathogen *Porphyromonas gingivalis* may be involved in the activation of glial cells, ultimately leading to neuroinflammation and impairment of memory function. In addition, not only does gingipain plausibly propagate among neurons in a manner reminiscent of infectious diseases, resulting in direct damage to Tau proteins, but it also activates human proteases associated with Tau proteins, thereby contributing to the pathological changes in Tau proteins observed in AD (32). Concurrently, diseased periodontal tissues secrete pro-inflammatory cytokines such as IL-1, IL-6, TNF-α, chemokines, and IL-8, along with bacteria that sustain the state of inflammation. These inflammatory mediators can enter circulation through the inflamed and dilated capillaries (35), effectively traversing the blood–brain barrier (BBB) (36) and gaining access to the central nervous system. Alternatively, they can disseminate to the brain via cranial nerves such as the olfactory nerve (37) and trigeminal nerve (38), prompting inflammatory cascades within the brain, ultimately culminating in neurodegeneration, brain atrophy, and cognitive decline (39). Collectively, these processes suggest a potential association with the development and advancement of AD, though further research is warranted.

Furthermore, individuals afflicted with AD possess the capacity to transmit their oral and gut microbiota to their non-AD counterparts, thereby exerting a cognitive impact on the recipients (40). This observation suggests that beyond the disrupted composition of the gut microbiota potentially contributing to AD pathogenesis via the intricate gut-brain axis (41), the oral microbiota can also influence perturbations in gut ecology through the “oral-gut-brain axis,” concurrently fostering conditions of neuroinflammation and neurodegeneration (38). Nevertheless, in contrast to the gut microbiota, the oral microbiota possesses a more direct and expeditious route to the brain, as it can readily breach the blood–brain barrier and initiate neuroinflammation (32). Animal studies have found that inducing periodontitis in mice simultaneously leads to progressive cognitive impairments. This phenomenon may be associated with an imbalance in the oral and gut microbiota triggered by the periodontal-related saliva microbiome. This imbalance, in turn, activates the LPS/TLR4/MyD88/NF-κ B signaling pathway, ultimately resulting in the disruption of the intestinal barrier and blood–brain barrier (42, 43). Consequently, based on current observations, it is tentatively postulated that the oral microbiota might potentially play a pivotal role in the initiation and progression of AD. This hypothesis arises from its perceived capacity to disseminate swiftly via hematogenous and neural routes, potentially facilitating its penetration into the cerebral domain. However, further research is needed to confirm this association (see Figure 1).

## 3 The potential application of oral microbiota in the prevention and treatment of AD

### 3.1 Early diagnosis

Recognizing the irreparable nature of the pathological changes attributed to AD, given its irreparable pathological changes, the early diagnosis of the disease holds crucial prognostic significance. However, Alzheimer’s Disease International (ADI) reports that globally, up to 75% of dementia cases go undiagnosed, with this number potentially rising to 90% in certain low- and middle-income countries, and it maintains a comprehensive early diagnosis strategy for AD that involves the synergistic use of cognitive testing, confirmatory scans, cerebrospinal fluid (CSF) analysis, and the incorporation of emerging biomarkers (7).

Since Klunk and colleagues first demonstrated 15 years ago the use of amyloid-β PET tracer Pittsburgh Compound-B (PIB) to directly visualize the pathophysiology of AD in the human brain (44), Aβ PET (45), and tau PET (46) have also begun to be gradually employed for early diagnosis of AD. Despite being available in high-income countries, the utility of PET and SPECT screening methods for identifying underlying causes of dementia is still limited due to cost and accessibility constraints. This implies that many regions globally are unable to fully utilize these advanced imaging techniques. Therefore, the search for more cost-effective and readily available screening methods to aid in early dementia diagnosis becomes crucial in achieving equitable distribution of healthcare resources worldwide.

While there is currently insufficient empirical evidence to support specific routine blood tests, recent research has demonstrated the feasibility of measuring blood biomarkers associated with AD, such as amyloid-β (Aβ), tau protein, phosphorylated tau (p-tau), and neurofilament light chain (NfL) in plasma (47). Furthermore, studies have shown a correlation between periodontitis and cognitive decline, potentially linked to overexpression of blood biomarkers for AD, such as p-tau and Aβ 1–40 (48).

Moreover, alterations in pro-inflammatory cytokine levels may hold diagnostic value for early identification of AD. Investigations conducted on AD patients reveal heightened levels of specific IgG antibodies directed against periodontal bacteria, notably exceeding those observed in non-AD counterparts, over the decade preceding the onset of the disease (49). However, a long-term follow-up study was carried out on individuals participating in the National Health and Nutrition Examination Survey III (NHANES III) from 1988 to 2019. The results indicated that the IgG antibody cluster of periodontal microbiota could not serve as a predictor for AD mortality (22). Additionally, a prospective longitudinal study has indicated that elevated levels of TNF-α in the blood plasma, as well as the presence of antibodies against periodontal pathogens, may be associated with the development or progression of AD, and these factors may contribute to improving the clinical diagnosis of AD (50).

AD patients exhibit heightened circulating levels of IL-1β, IL-2, IL-6, IL-18, α-1 antichymotrypsin, and C-reactive protein (CRP) which may serve as potential biomarkers for the diagnosis of AD (51). Notably, elevated CRP and IL-6 concentrations have been associated with a 45 and 32% increased risk of multi-etiological dementia, respectively, underscoring the potential involvement of inflammation in the onset and progression of this intricate form of dementia (52).

### 3.2 Treatment

#### 3.2.1 Basic periodontal therapy

In the context of AD, elderly individuals often encounter the challenge of maintaining proper oral hygiene, leading to an augmented colonization of bacteria within the oral cavity (53). Scaling and root planning (SRP) represent the gold standard approach for addressing periodontitis, encompassing a meticulous procedure designed to eradicate both supragingival and subgingival dental plaque and calculus. Through targeting and elimination of these sources of infection, SRP not only diminishes the abundance of pathogenic bacteria and inflammatory mediators in the oral milieu but also promotes oral health (54). The implementation of SRP can significantly alleviate the burden of detrimental microorganisms and ameliorate oral inflammation, potentially endowing noteworthy therapeutic benefits for individuals afflicted with AD. Particularly noteworthy, individuals who neglect to accord priority to fundamental periodontal treatment face a substantially heightened risk of developing AD (55). Treating periodontitis has been suggested to potentially improve AD-related brain atrophy, with both periodontal treatment and subsequent maintenance therapy influencing imaging biomarkers and showing promising efficacy in treating AD-related brain atrophy (56). Interestingly, in cases of mild AD, the integration of periodontal therapy has shown potential for improving oral health indicators, enhancing overall quality of life (57) and may also hold promise as a possible approach to support cognitive function and promote brain health.

Unfortunately, individuals with dementia face increasing challenges in accessing essential dental healthcare as their cognitive function declines (58). Recent studies have highlighted concerning issues surrounding oral care interventions for hospitalized elderly patients, citing the high cost associated with dental healthcare and the limited health benefits considering their shorter life expectancy (59). Therefore, when it comes to treating periodontal disease in patients with AD and improving oral hygiene, it is important to consider the feasibility and appropriateness of treatment measures. Additionally, increasing awareness of health interventions in this area is crucial to ensure that elderly patients receive the necessary oral care services they require.

#### 3.2.2 Antibiotics

In light of the association between alterations in the oral microbiome and AD, the administration of antibiotics has emerged as a potential avenue to ameliorate AD symptoms (60, 61). Despite achieving a substantial reduction in bacterial populations through gingival curettage and subgingival scaling, the primary challenge for long-term effectiveness in managing periodontitis lies in controlling the regrowth of oral microbiota (62). Notably, tetracycline antibiotics such as doxycycline and minocycline have garnered attention due to their capability to traverse the blood–brain barrier (BBB) (63) and their diverse effects within the central nervous system. These effects include the inhibition of matrix metalloproteinases (MMPs), scavenging of reactive oxygen species (ROS), anti-apoptotic properties, anti-inflammatory effects, inhibition of protein aggregation, and preservation of mitochondrial function (64). Consequently, these antibiotics have been identified as potential therapeutic modalities for AD. However, the dosages of medications required to impact and improve cognitive status are much higher compared to standard doses. For instance, minocycline doses ranging between 20–100 mg/kg have been proposed to achieve the desired neuroprotective effects, which significantly exceeds the dosages commonly employed for inflammation and infections (typically around 3 mg/kg/day) (65). However, long-term use of such high doses of antibiotics can disrupt the gut microbiota, leading to gastrointestinal adverse reactions (66), and bacterial resistance. Whether there is a way to control both periodontal inflammation and brain inflammation, reduce the dosage of minocycline, and therefore mitigate potential side effects.

Consequently, an emerging strategy that involves the synergistic utilization of scaling and root planning (SRP) with the integration of local drug delivery systems (LDDSs) or sustained-release drug delivery techniques (67). Notably, research has demonstrated that patients receiving adjunctive treatment of scaling and root planning utilizing minocycline hydrochloride microspheres displayed a notable reduction in both counts of periodontal pathogens and levels of periodontal attachment loss, surpassing the outcomes observed in the control group (68). Nanocarriers offer advantages in enhancing drug solubility and dissolution rate, improving oral bioavailability, and reducing side effects and dosing frequency (69), and they may have a potential role in improving cognitive function in AD (70). The research findings by Kashi et al. indicate that the use of nanocarriers to load minocycline can reduce the minimum inhibitory concentration (MIC) and minimum bactericidal concentration (MBC) by half, compared to using minocycline alone, thereby reducing the required drug dosage (71). Animal studies have demonstrated that minocycline loaded into polymer nanoparticles can alleviate neuroinflammation in mice with spinal cord injury (72). Combining local drug delivery systems and nanocarrier technologies to use antibiotics for treating AD symptoms may be a potentially effective therapeutic approach. The application of these new technologies holds the potential to enhance treatment efficacy and minimize the side effects and resistance issues associated with antibiotics. Nevertheless, more scientific evidence is needed to support this perspective.

#### 3.2.3 Probiotics

Probiotics, as defined by the World Health Organization (2012), pertain to “viable microorganisms that, when administered in appropriate quantities, confer health benefits to the host” (73). Notably, in 2012, Sugano introduced the concept of “biofilm control,” which encompasses the utilization of probiotics and vaccines to eradicate pathogenic bacteria (74). As a result, probiotic adjunctive therapy has emerged as a prospective method to prevent or treat intestinal dysbiosis and optimize the therapeutic effects of AD medication by modulating the gut-brain axis (75, 76).

Of note, these probiotic species are also important members of the oral-gut-brain axis, playing critical roles in the intricate ecosystem of human microbiota. The Lactobacillaceae and Bifidobacteriaceae families have gained prominence as extensively investigated probiotic species within the realms of dentistry and medicine. These microbial families assume critical roles as integral constituents of the human oral, gastrointestinal, and urogenital microbiota (77). Additionally, select strains of Lactobacillus and Bifidobacterium have exhibited the ability to modulate brain function through epigenetic regulatory mechanisms (78), which could potentially lead to the attenuation of inflammatory processes. Particularly noteworthy is the *Lactobacillus casei* strain ML2018, which has demonstrated the potential to counteract the pro-inflammatory effects of lipopolysaccharides and mitigate the release of cytotoxic molecules, including nitric oxide (79). Intriguingly, animal models have revealed that oral probiotic administration might have the potential to restore glucose homeostasis in mouse models of AD by potentially exerting influences upon the intricate ecosystem of the gut microbiota (80) and potentially modulate the microbiota-gut-brain axis and improve memory (81). Clinical trials have demonstrated that supplementation with probiotics can promote psychological flexibility and reduce stress in healthy older adults by modulating the gut microbiota, and may improve cognitive function and mood (82).

Furthermore, probiotics have emerged as a promising adjunctive therapeutic intervention in the management of periodontal diseases. Currently, commercially available *Lactobacillus reuteri* lozenges have been successfully commercialized and effectively utilized as a supportive treatment modality in periodontal therapy, yielding favorable outcomes (83). These lozenges have demonstrated the potential to mitigate the dysbiotic subgingival microbial environment after periodontal interventions and effectively modulate the microbial composition within the depths of periodontal pockets, including the challenging-to-access furcation areas (84). These contributions significantly contribute to the long-term preservation of the therapeutic efficacy achieved through periodontal therapy. Particularly for elderly patients with compromised oral hygiene abilities, the use of probiotics offers a user-friendly and low-risk approach. As a standalone therapeutic modality, it holds considerable prospects for incorporation into oral healthcare practices. Interestingly, data from a longitudinal study spanning 60 days showcased that monotherapy administration of *Lactobacillus reuteri* lozenges for periodontitis exerts selective antimicrobial activity against pathogenic organisms inhabiting the periodontal niche. This leads to significant reductions in pocket depth, attachment loss, and plaque accumulation in untreated molars with deep periodontal pockets. Notably, it also yields remarkable effects in the resolution of fistulas and the maintenance of prolonged equilibrium in the subgingival microbial community (85).

#### 3.2.4 Biopharmaceuticals

Cathepsin B (CTSB) is a highly potent lysosomal protease that plays a crucial role in the pathogenesis of the destructive inflammatory cascade orchestrated by lipopolysaccharide (LPS). The disruption of the integrity of the blood–brain barrier may has been implicated in the etiology of LPS derived from *Fusobacterium nucleatum* (86). Notably, an animal experiment revealed that prolonged exposure to LPS in middle-aged mice may induce pathological changes associated with AD, with CTSB playing a mediating role in this process (87). This implies that CTSB holds promise as a therapeutic target for preventing cognitive decline in AD related to periodontitis. Interestingly, studies utilizing genetic knockout of the CTSB gene have revealed its capacity to mitigate memory loss and neuroinflammation induced by *Porphyromonas gingivalis* infection. Additionally, the administration of CTSB inhibitors has been shown to effectively dampen the inflammatory response triggered by LPS thereby displaying the potential to enhance cognitive function in individuals affected by AD (88). In summary, a comprehensive understanding of the intricate interplay among CTSB, LPS, and the disruption of the blood–brain barrier has shed light on the utilization of CTSB inhibitors and the modulation of CTSB gene expression as prospective strategies to combat cognitive decline in chronic periodontitis-associated AD.

Gingipains, as the primary virulence factors synthesized by *Porphyromonas gingivalis*, are cysteine proteases that exert a fundamental role in host colonization, tissue degradation, evasion of host immune responses, and acquisition of vital nutrients, such as iron (89). The administration of systemically delivered gingipain inhibitors has emerged as a potent strategy for reducing the population density of *Porphyromonas gingivalis* and mitigating neuroinflammation, thereby providing neuroprotection to hippocampal neurons (32). An important advantage associated with gingipain inhibitors, in comparison to broad-spectrum antibiotics, is their specific targeted action against *Porphyromonas gingivalis*, effectively mitigating the imminent risk of antibiotic resistance development.

APOE4 (apolipoprotein E4) is a 34 kDa plasma lipoprotein that has recently been found to have a potential association with both AD (90) and periodontitis (91). The autopsy report not only presents evidence of a positive correlation between APOE4 allele and sporadic AD (92), but also observes an association between APOE genotypes and β-amyloid deposition in the cerebral cortex, even in elderly participants without AD (93). The relationship between periodontitis and AD may be explained by the presence of the APOE4 gene. A case-control study using tooth loss as a surrogate measure for periodontitis has shown an association between mild cognitive impairment (MMI) and the number of teeth lost in individuals carrying the APOE4 allele (94). Furthermore, *Porphyromonas gingivalis* may enter the brains of ApoE−/− mice, leading to complement activation and damage to surrounding neurons (95). This neuroinflammatory mechanism induced by *Porphyromonas gingivalis* could potentially generate neurotoxic fragments of APOE in AD brains. The detection of *Porphyromonas gingivalis* in the central nervous system suggests a potential correlation between the development of AD and genetic variations associated with the host immune response, including genes such as TREM2 (96), TLR4 (97), CR1 (98), and NLRP3 (99). To devise more personalized prevention and treatment strategies, future research can concentrate on elucidating internal changes and genetic variations occurring within the host.

### 3.3 Prevention

#### 3.3.1 Oral hygiene maintenance

As AD progresses, the cognitive decline experienced by patients adversely affects their ability to uphold oral hygiene practices and limits their access to dental care (100), resulting in a notable decline in their oral health status (101). Research has indicated that only 13% of adults aged 50 and above in Organization for Economic Co-operation and Development (OECD) member countries engage in weekly oral hygiene practices (102). Thus, healthcare professionals assume a crucial role in delivering comprehensive oral education and conducting regular follow-up visits to evaluate the oral condition of patients, ensuring effective periodontal maintenance (103). The implementation of the 5S methodology, comprising sorting (Seiri), straightening (Seiton), sweeping (Seiso), standardizing (Seiketsu), and sustaining (Shitsuke), is gradually integrated into oral hygiene practices (96). This approach assists healthcare providers in enhancing hygiene standards and work efficiency while caring for elderly patients with AD, which potentially yields improved overall oral health outcomes (104).

Considering the impact of cognitive impairments on oral health and dental utilization (105), a longitudinal study conducted in China unveiled the challenges faced by elderly individuals with dementia in maintaining sufficient oral hygiene habits, resulting in suboptimal oral health status and an elevated vulnerability to dental caries. These difficulties can be attributed to obstacles encountered during toothbrushing (53). In comparison to traditional manual toothbrushes and dental floss, the adoption of powered toothbrushes, water flossers, or Collis curve brushes has emerged as a promising intervention, providing simplified oral care procedures and enhanced plaque removal efficacy, thereby promoting improved oral hygiene practices (106).

#### 3.3.2 Vaccine

The research findings present evidence that the release of OMVs by *Porphyromonas gingivalis* triggers the activation of the NLRP3 inflammasome, leading to neuroinflammation, tau phosphorylation, and cognitive impairment in mice. This cascade of events has the potential to have a latent impact on the progression of AD (107), although the exact extent of its influence is still uncertain. Notably, the gingival cytoplasmic membrane vesicles possess nanoscale dimensions and exhibit remarkable immunogenicity, adaptability, and immunocyte uptake, thereby displaying functional intercellular interactions. These unique characteristics position them as a promising avenue for the development of vaccines and targeted drug delivery systems aimed at suppressing the release of bacterial virulence factors (108). Currently, extensive research and development efforts are underway to formulate vaccines tailored specifically to combat periodontal pathogens, particularly *Porphyromonas gingivalis* (109). Anticipated advancements in vaccine development technologies hold promise for the creation of an expanded repertoire of vaccines targeting a diverse array of periodontal pathogens in the foreseeable future. These vaccines have potential in effectively preventing both oral diseases induced by oral bacteria and systemic diseases. Furthermore, the development of vaccines against periodontal pathogens is expected to yield improvements in global oral health, thereby exerting a impact on overall human health and well-being.

#### 3.3.3 Healthy lifestyle

Periodontitis and AD bear resemblances in terms of multiple shared risk factors, including age, obesity, diabetes, psychological stress, smoking, alcohol consumption, and education level. Consequently, implementing measures to modify lifestyle habits in these domains can serve as an effective strategy in concurrently preventing and ameliorating both periodontitis and AD.

Immunosenescence and inflammation are intricately interconnected processes that synergistically contribute to the pathogenesis of periodontitis and AD (110). Insufficient physical activity, imbalanced dietary patterns, and excessive nutrient intake can elicit inflammatory responses and oxidative stress, disrupting crucial metabolic pathways and ultimately predisposing individuals to obesity and diabetes (111). Therefore, promoting a comprehensive diet and physically active lifestyle among older individuals is of paramount importance to bolster the immune system, mitigate the risks associated with obesity and diabetes, and foster a vibrant aging process. Additionally, dietary modifications have the potential to exert positive effects on both oral and gut microbiota, thereby imparting further benefits to overall health.

The Mediterranean-style diet is widely recognized as an exemplary anti-inflammatory dietary pattern that confers protective effects against age-related risk factors for disease. It is characterized by a generous consumption of whole grains, fruits, vegetables, legumes, and olive oil while emphasizing moderation in dairy products and alcohol, and limiting meat intake (112). Animal studies have demonstrated that the primary component of the Mediterranean diet, oleic acid, exhibits remarkable anti-inflammatory properties, and also contributes to the resolution of inflammation, thereby ameliorating tissue damage resulting from the host response to oral bacterial ingestion in mice (113). Mild cognitive impairment (MCI) represents an early stage of AD pathology and necessitates non-pharmacological interventions, including dietary and nutritional modifications. Engaging in aerobic exercises and other physical activities has shown an inverse correlation with the reduction of AD-related impairments (114). For individuals aged 45 and above with MCI progressing towards AD, adopting the Mediterranean-style diet and incorporating regular 3 to 5 days of medium-to-high-intensity physical exercise per week can yield improvements in overall cognitive function among MCI patients (115).

Stress emerges as a risk factor in the etiology of depression, although the direct causal link between stress and AD as a risk factor remains to be definitively established. Nevertheless, clinical investigations have revealed a remarkable finding: individuals suffering from depression face a significantly heightened risk, exceeding threefold, of developing AD (116). The impact of stress is mediated through the activation of apoptotic mechanisms in the astrocytes of the hippocampus and prefrontal cortex, thereby may exacerbating the shared pathological characteristics of depression and AD (117). Furthermore, the intricate interplay between stress and the diversity of the microbiome may exert a regulatory influence on stress responses and anxiolytic effects, ultimately shaping the potential progression of AD. Enlightening research suggests that the administration of probiotics and short-chain fatty acids (SCFAs) may promote cognitive restitution and enhance neuropsychiatric well-being by suppressing stress-induced cortisol release in chronically distressing socio-psychological environments (118). Simultaneously, the impact of potentially addictive substances, such as tobacco, alcohol, and/or anesthetics, may influence both host reactions and the microbiome, thereby possibly impinging upon the trajectory of AD (119).

The extensive utilization of music for relaxation and stress reduction purposes is widely recognized. By stimulating both the sensory and cognitive centers of the brain, music has the potential to enhance attention, and concentration, and foster creativity. Consequently, the application of music therapy in the context of AD has garnered t attention from researchers and practitioners. A growing body of evidence supports the effectiveness of music therapy in mitigating behavioral disturbances, anxiety, and agitation among individuals with AD (120). Additionally, classical music has been observed to stimulate salivary secretion and facilitate the production of salivary nitrite, with potential implications for modulating oral function (121). Salivary nitrite, derived from nitrate, can undergo further reduction to nitric oxide (NO) and other nitrogen oxides within the bloodstream and tissues (122). Salivary nitrite exerts gastroprotective effects, while the combination of nitrite-rich human gastric fluid and saliva has been shown to inhibit the proliferation of *Escherichia coli* and *Candida albicans* (123). Interestingly, patients with periodontal disease exhibit notably higher concentrations of total nitrate and nitrite in their saliva, suggesting a potential link to the host’s defense mechanisms and providing an additional layer of protection against these infectious conditions (124). Although further investigation is needed to elucidate individual variations in treatment response and underlying mechanisms, music therapy represents a cost-effective and non-pharmacological intervention that holds promise in enhancing the psychophysical well-being of individuals with AD and exerting favorable influences on oral health.

## 4 Conclusion

There may be a potential reciprocal relationship between oral dysbiosis and AD. The recognition of oral bacteria as a novel perspective for enhancing AD provides new therapeutic avenues for oral healthcare professionals. For the purpose of prevention, healthcare practitioners can consider reinforcing oral health education among older adults and promoting regular oral examinations. It is worth noting that there is a tendency to overlook oral healthcare in this age group, and the associated costs can sometimes be discouraging for patients. However, it is important to address these challenges and find ways to raise awareness and facilitate access to affordable oral healthcare services. For treatment, a combination of routine periodontal therapy and adjunctive use of antibiotics and probiotics could be considered. Non-pharmacological interventions, which may include adopting a healthy diet and lifestyle, have been suggested to potentially enhance cognitive function, neurological and mental well-being, and overall quality of life among AD patients. Despite the immense potential and promising prospects of harnessing oral bacteria for AD prevention and treatment, several unresolved challenges persist. Particularly, refining methods for early detection of AD through emerging blood biomarkers remains incomplete. Moreover, further investigation is warranted to determine the optimal dosage of antibiotics and probiotics, as well as potential side effects. The utilization of oral bacterial vaccines offers a promising approach to AD treatment, but necessitating substantial research and development endeavors are necessary to pave the way for effective AD prevention and management. In the future, further long-term, high-quality scientific research and rigorous clinical trials are needed to validate its effectiveness and safety, thus providing more effective means for the prevention and treatment of AD.

## Author contributions

MZ: Writing – original draft. NM: Literature search. ZY: Data curation. XL: Language modification & editing. YJ: Study design & manuscript review & editing. All authors contributed to the article and approved the submitted version.
